# Solvothermal synthesis of lanthanide-doped CaF_2_upconversion nanoparticles using liquid-solid-solution approach

**DOI:** 10.1038/s41598-025-17706-7

**Published:** 2025-10-03

**Authors:** Kapil S. Janbandhu, V. B. Pawade, Anil M. Pethe, S. J. Dhoble

**Affiliations:** 1 Department of Physics, R.T.M. Nagpur University, Nagpur, 440033 India; 2 Department of Physics, Laxminarayan Innovation Technological University, Nagpur, 440033 India; 3 Datta Meghe College of Pharmacy, Datta Meghe Institute of Higher Education & Research, Sawangi (Meghe), Wardha, 442001 India

**Keywords:** CaF_2_, UCNPs, LSS, Solvothermal, Fluoride, Nanoparticles, Nanoscience and technology, Physics

## Abstract

Upconversion nanoparticles (UCNPs) capable of emitting pure green, red, blue, and white emissions have grabbed a lot of attention for bioimaging, security printing, and optoelectronic devices. Ho^3+^/Yb^3+^, Er^3+^/Yb^3+^, Tm^3+^/Yb^3+^, and Er^3+^/Tm^3+^/Yb^3+^ double- and triple-doped CaF_2_ nanoparticles (NPs) were synthesized by the solvothermal method. The nanoparticleswere characterized by XRD, FTIR, HR-TEM, Raman, and photoluminescence spectroscopy. Under 980 nm, UCNPs emit red, green, blue, yellow, and white emissions by the appropriate doping of lanthanide ions.The CIE-1931 chromaticity study was carried out to determine the color coordinate and color purity of the UCNPs. The color purity of red, green, blue, and yellow emissions exceeds 90%, showing the excellent chromatic performance. The energy transfer mechanismin the sensitizer Yb^3+^ions withthe activators Ho^3+^, Er^3+^, and Tm^3+^ was successfully studied. Er^3+^/Yb^3+^ co-doped CaF_2_ UCNPs were used to study the potential scope in latent fingerprint detection and security applications.

## Introduction

Lanthanide (Ln)-doped upconversion nanoparticles (UCNPs) are a unique class of luminescent materials that absorb multiple low-energy photons and convert them into high-energy photons. Upconversion (UC) is a photon-assisted anti-Stokes process that can exhibit a large wavelength shift, often exceeding 10 kT, depending upon the specific emission characteristics of the doped Ln^3+^ ions. It is significantly greater than that of the thermally excited (phonon-assisted)anti-Stokes process, which has a shift of only a few kT. This is due to sequential absorption of two or more low-energy photons by the Ln^3+^ ions, which makes it very advantageous for applications^1^. Recent literature highlights the significance of UCNPs in various fields, including anticounterfeit, forensic science, thermometry, displays, optoelectronic devices, and biomedical science^2–7^. Especially in the biomedical field, UCNPs offer the unique ability to provide localized UV, visible, or NIR emissions at the tissue sites due to the deep penetration depth of exciting NIR laser radiation, an advantage that is not achievable with the traditional downshifting (or downconversion) nanoparticles that require low-penetrable UV/visible excitation.The UCNPs, in combination with a photosensitizer (PS) molecule, can be used in photodynamic therapy, which is considered less invasive than radiotherapy and chemotherapy. First, NIR light is converted into visible light using UCNPs that activate the PS molecule. The activated PS transfers energy to neighboring oxygen and generates singlet oxygen^1^O_2_) or reactive oxygen species (ROS), which can further destroy the tumor and ultimately contribute to cancer cell death. NIR-to-UV or NIR-to-Visible UCNPs can be employed in targeted drug delivery systems, where the therapeutic drugs are loaded onto the UCNPs and shielded by light-responsive compounds. Upon NIR excitation, the UCNPs emit UV or visible light that overlaps with the absorption of the light-sensitive compound. This triggers a photoreaction, resulting in the controlled release of the drug specifically at the targeted site^8,9^. NIR-to-NIR or NIR-to-visible (red and green) UCNPs are promising for bioimaging and detection applications due to their deep tissue penetration, low background autofluorescence, and high photostability^10^.

Hosts such as NaREF_4_, LiREF_4_, KREF_4_, and REF_3_ (RE = rare earth) have been attracted for the UCNPs; however, rare earth-free hosts such as MF_2_ (M = alkaline earth metals) also present compelling candidates for UCNPs due to cost effectiveness, high bandgap, chemical stability, low phonon energy, and similar ionic radii for lanthanide dopants. CaF_2_ is a well-known host for UCNPs that shows great potential for biomedical applications due to its biocompatibility and optical transparency. Balabhadra et al.^11^ prepared citrate-cappedEr^3+^/Yb^3+^ co-doped CaF_2_, SrF_2_, BaF_2_ UCNPs by the hydrothermal method using sodium citrate. Pedroni et al.^12^ prepared cubic-shaped oleate-capped CaF_2_ NPs doped with Er^3+^/Yb^3+^, Tm^3+^/Yb^3+^, and Ho^3+^/Yb^3+^ ions by a one-pot hydrothermal method. Wang et al.^13^ reported bright green emitting CaF_2_: Er^3+^/Yb^3+^ sub-10-nm monodispersed UCNPs by aliquid-solid-solution (LSS) strategy. They compared the upconversion luminescence properties with well-known NaYF_4_:Er^3+^/Yb^3+^ and found that CaF_2_ can be a promising host for UCNPs. Dong et al.^14^ reported CaF_2_: Tm^3+^/Yb^3+^ NIR-to-NIR emitting UCNPs prepared by the hydrothermal method using citrate as a capping agent and found scope in fluorescent bioimaging applications. Misiak et al.^15^ prepared CaF_2_: Tm^3+^/Yb^3+^ UCNPs by the thermal decomposition method and demonstrated application in labelling and imaging of *Candida albicans* cells. Zhou et al.^16^ reported CaF_2_:Yb/Tm/Ho nanoparticles for white light emission and enhanced emission by NaYF_4_:Yb shell growth.In the current study, we report green, yellow, red, blue, and white emitting CaF_2_ UCNPs doped with Ho^3+^/Yb^3+^, Er^3+^/Yb^3+^, Tm^3+^/Yb^3+^, and Er^3+^/Tm^3+^/Yb^3+^ usingthe solvothermal method via a liquid-solid-solution approach. Further, we have discussed the scope of UCNPs for latent fingerprint detection and anticounterfeit applications.

### Experimental

Calcium Nitrate Tetrahydrate (Ca(NO_3_)_2_.4H_2_O, Loba Chemie, 99%), Sodium Fluoride (NaF, Loba Chemie, 99%), Holmium Oxide (Ho_2_O_3_, Loba Chemie, 99%), Erbium Oxide (Er_2_O_3_, Himedia, 99.99%), Thulium Oxide (Tm_2_O_3_, Ottokemi, 99.99%), Ytterbium Oxide (Yb_2_O_3_, SRL, 99.9%), Sodium Hydroxide (NaOH, Loba Chemie, 98%), Nitric Acid (HNO_3_, Fisher Scientific, 70%),Oleic Acid (C_17_H_33_COOH, Loba Chemie, Extra Pure), Cyclohexane (C_6_H_12_, Fisher Scientific, 99%), Chloroform (CHCl_3_, Sigma-Aldrich, 99%), Ethanol, andDistilled Water reagents and solvents were used. The rare earth oxides, i.e.,Ho_2_O_3_, Er_2_O_3_, Tm_2_O_3_, and Yb_2_O_3_, were dissolved in nitric acid at elevated temperature and evaporated and finally dissolved in distilled water to get Ho(NO_3_)_3_, Er(NO_3_)_3_, Tm(NO_3_)_3_, and Yb(NO_3_)_3_solutions, respectively.

The Ln^3+^-doped CaF_2_UCNPs were synthesized by the liquid-solid-solution (LSS) solvothermal approach^17^. For the synthesis of CaF_2_ nanoparticles, 12.6 mLof Oleic Acid (OA), 36 mLof Ethanol, and 0.4 g of NaOH were mixed and magnetically stirredfor 1 h to get solution-1. In another beaker, 6 mmol of Ca(NO_3_)_2_.4H_2_O is dissolved in 10 mLof distilled water to get a metal nitrate solution and is namedsolution-2. Similarly, 12 mmol of NaF was dissolved in 5 mL of distilled water to get aNaF solution and named it solution-3. Solution-2 is then added to solution-1 and stirred for 15 min. After 15 min, solution-3 is also added and stirred for 1 h. All the mixing was done at room temperature. After 1 h, the solution is transferred to a 100 mL Teflon-lined autoclave, the autoclaveis sealed tightly, and placed in a hot air oven for 24 h at 160 °C. After 24 h, the autoclave system was allowed to cool to room temperature naturally. The OA-capped nanoparticles that had settled were collected from the bottom of the Teflon container. The nanoparticles were dispersed in small amounts of cyclohexane, reprecipitated by the addition of ethanol, and collected by centrifugation. This process is repeated several times to remove an excess amount of OA. The nanoparticles were then either dried at 70 °C or redispersed in cyclohexane. For the synthesis of 2 mol% Ho^3+^/20 mol% Yb^3+^, 2 mol% Er^3+^/20 mol% Yb^3+^, 2 mol% Er^3+^/40 mol% Yb^3+^, 0.5 mol% Tm^3+^/20 mol% Yb^3+^, and2 mol% Er^3+^/0.5 mol% Tm^3+^/20 mol% Yb^3+^ co-doped CaF_2_ nanoparticles, a stoichiometric amount of the corresponding rare earth Nitrates was added while preparing solution-2.

The X-ray diffraction patterns of the samples were recorded in the 2θ range of 20° to 80° with a step size of 0.04°using Rigaku’s Miniflex 600 X-ray diffractometer with a CuKα X-ray radiation source having a wavelength of λ = 0.154 nm, operating at 15 mA of current and 40 kV of voltage. Fourier Transform Infrared spectroscopy was carried out using a Bruker, Germany, 3000 Hyperion Microscope with a Vertex 80 FTIR System. High-resolution transmission electron microscopy (HR-TEM) images were taken using aJEOL JEM 2100 F Field emission gun transmission electron microscope, operating at 200 kV of accelerating potential. Laser Raman spectrum was recorded using Horiba Jobin Yvon, HR800-UV confocal micro-Raman spectrometer, excited using 50 mW, 542 nm Laser source.The photoluminescencespectrum of pure CaF_2_ NPs was recorded using an F-5301 PC Spectrofluorophotometer using a50 W xenon lamp as the excitation source.Upconversion luminescence emission spectra were recorded using a Horiba Jobin-Yvon (Fluorolog-3) fluorescence spectrofluorometer equipped with a980 nm LASER under 980 nm, 4 mW/cm^2^ laser excitation. The CIE-1931 chromaticity diagram was plotted using Osram-Sylvania Color Calculator software.

## Results and discussion

### X-ray diffraction (XRD) analysis

**Fig. 1:**
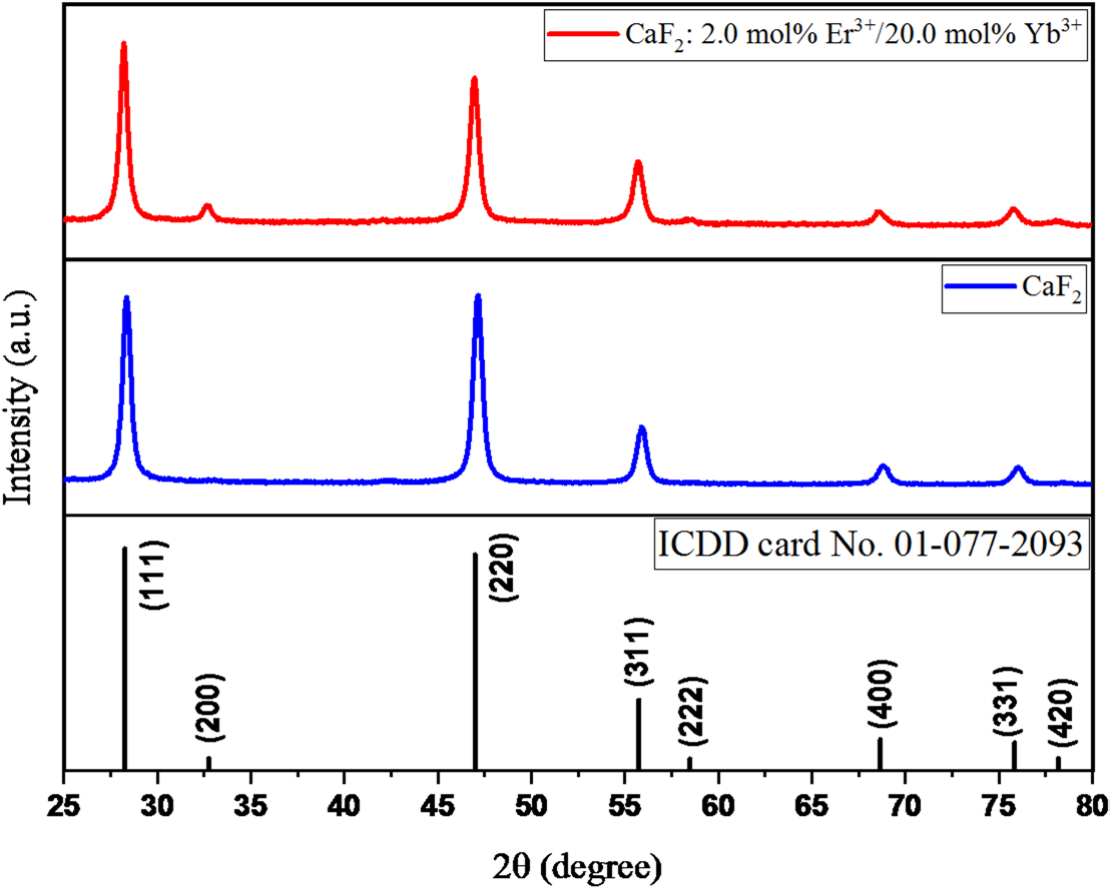
XRD patterns of pure CaF_2_ and CaF_2_: Er^3+^/Yb^3+^ UCNPs.

**Fig. 2:**
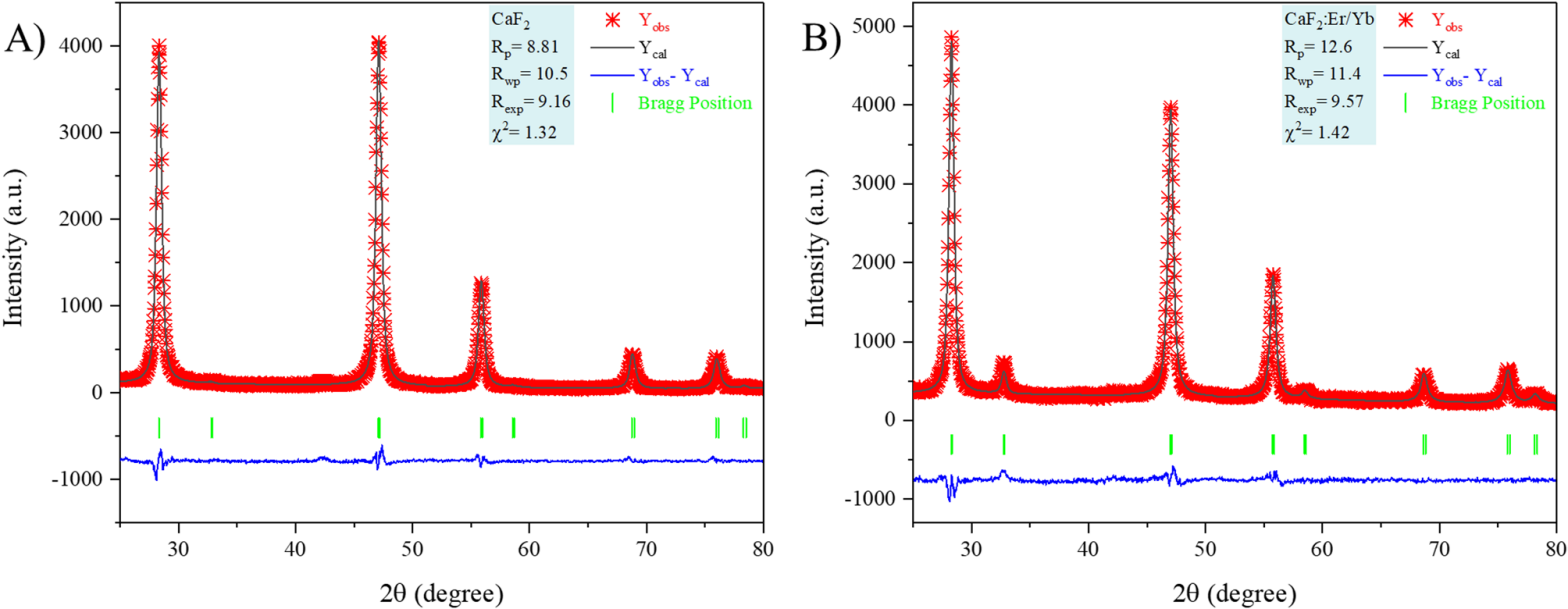
Rietveld refinement results of **(A)**pure CaF_2_ NPs and **(B)** CaF_2_: Er^3+^/Yb^3+^ UCNPs.

**Fig. 3:**
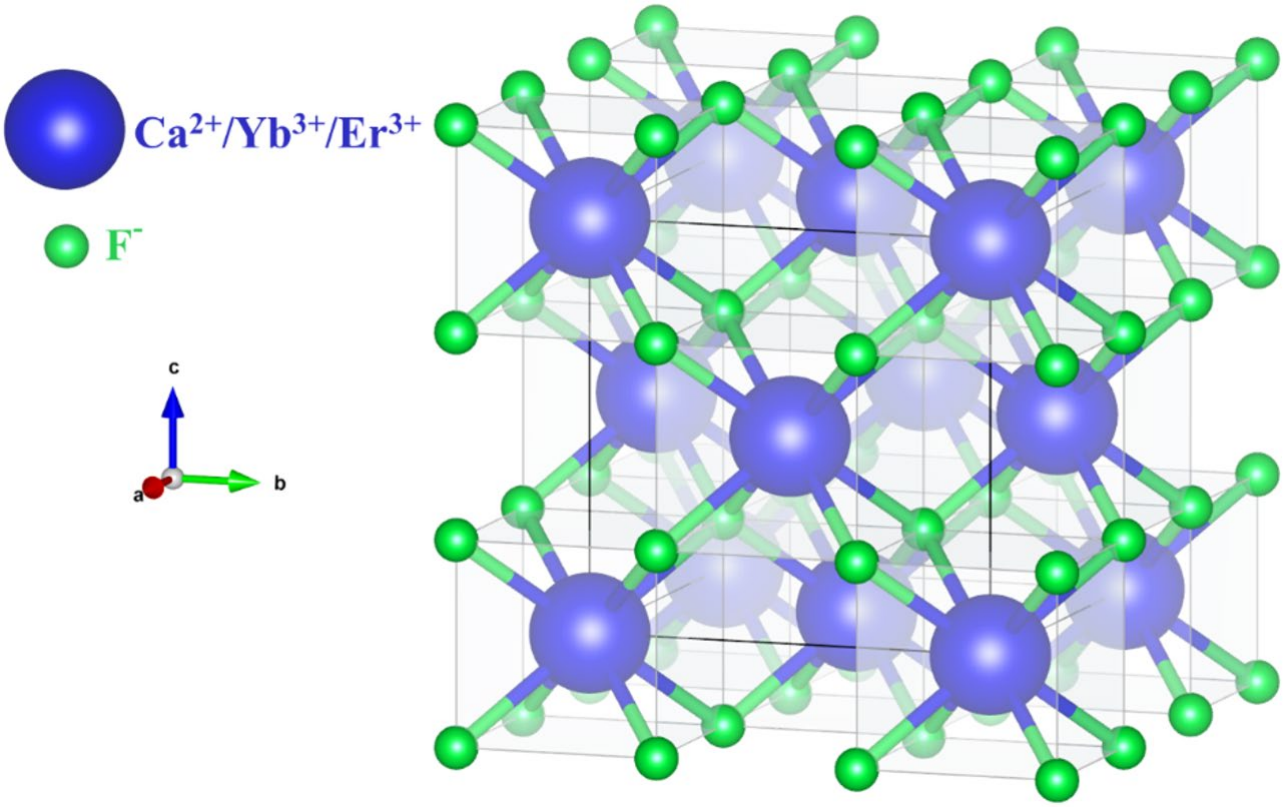
Crystal structure of CaF_2_: Er^3+^/Yb^3+^ UCNPs.

Figure 1 depicts the XRD pattern of undoped CaF_2_ and CaF_2_: Er/Yb nanoparticles with standard diffraction patterns(ICDD file no. 01-077-2093). Both experimental XRD patterns arewell-matched with the XRD standard data. CaF_2_ has a fluorite structure that crystallizes in the face-centered cubic (FCC) structure with the Fm-3 m (225) space group. Doping with higher concentrations of 20 mol% Yb^3+^ and 2 mol% Er^3+^ lanthanide ions does not result inany additional phase; therefore, Ca^2+^ ions are successfully substituted by Yb^3+^ and Er^3+^ ions. The intensities of diffracted peaks with 2θ angles of 32.7^o^ (200), 47^o^ (220), and 58.5^o^(222) are varied after doping as compared to undoped CaF_2_, this is because of the lattice distortion as ionic radii of Yb^3+^(0.99 Å) and Er^3+^(1.0 Å) ions are slightly different from Ca^2+^(1.12 Å)ions.The structural changes due to doping were investigated using Rietveld refinement analysis. Figure 2.showsthe observed pattern (Y_obs_), the calculated pattern (Y_cal_), the difference (Y_obs_- Y_cal_), and Bragg positions with reliability factors extracted from Rietveld refinements of Fig. 2. A).undoped- CaF_2_ andFig. 2. B).Er/Yb-doped CaF_2_ nanoparticles, respectively. Both the refined profiles show good fitting between calculated and observed patterns. Figure 3. depicts the refined crystal structure of CaF_2_: Er^3+^/Yb^3+^generated after Refinement. The metal ions (Ca^2+^/Yb^3+^/Er^3+^) occupy ‘4a’ sites, i.e., corner and face-centered positions, and fluorine ions (F^−^) occupy ‘8c’ sites of the fluorite structure. The cell parameters for undoped CaF_2_ and CaF_2_: Er^3+^/Yb^3+^ are a = 5.4566 Å and 5.4661 Å, respectively. The cell parameters are expected to decrease after doping, as the Ca^2+^ ions are larger than the substituting Yb^3+^ and Er^3+^ ions; however, contrary to expectations, the cell parameters slightly increased after doping. This increase in cell parameters is due to the entry of F^−^ ions in interstitial sites of the fluorite structure. These F^−^ ions act as charge compensators to overcome the charge imbalance formed after doping of trivalent lanthanide ions to substitute divalent Ca ions. This increase in cell parameters after doping of Yb^3+^ in CaF_2_ was also reported previously^18^.

The average crystallite size of CaF_2_ without doping and with doping of Er/Yb was estimated by using the Scherrer formula-

$$\:D=\:\frac{k\lambda\:}{\beta\:\text{cos}\theta\:}$$

Where D is the crystallite size, k is the shape factor (approximately k = 0.9), λ is the wavelength of X-ray radiation (λ = 0.154056 nm), β is the full width at half maximum (FWHM) of the XRD peak, and θ is Bragg’s angle. The crystallite size ofundoped CaF_2_ and Er/Yb-doped CaF_2_ was found to be the same, approximately 15 nm. However, on the peak broadening, the Scherrer formula does not consider the microstrain, which can also affect the peak broadening. This can be resolved by the Williamson-Hall (W-H)equation, which includes peak broadening due to crystallite size (D) and microstrain (ε) -

$$\:{\upbeta\:}\:\text{c}\text{o}\text{s}\:{\uptheta\:}=\frac{\text{k}{\uplambda\:}}{\text{D}}+4\:{\upepsilon\:}\:\text{s}\text{i}\text{n}\:{\uptheta\:}$$

**Fig. 4:**
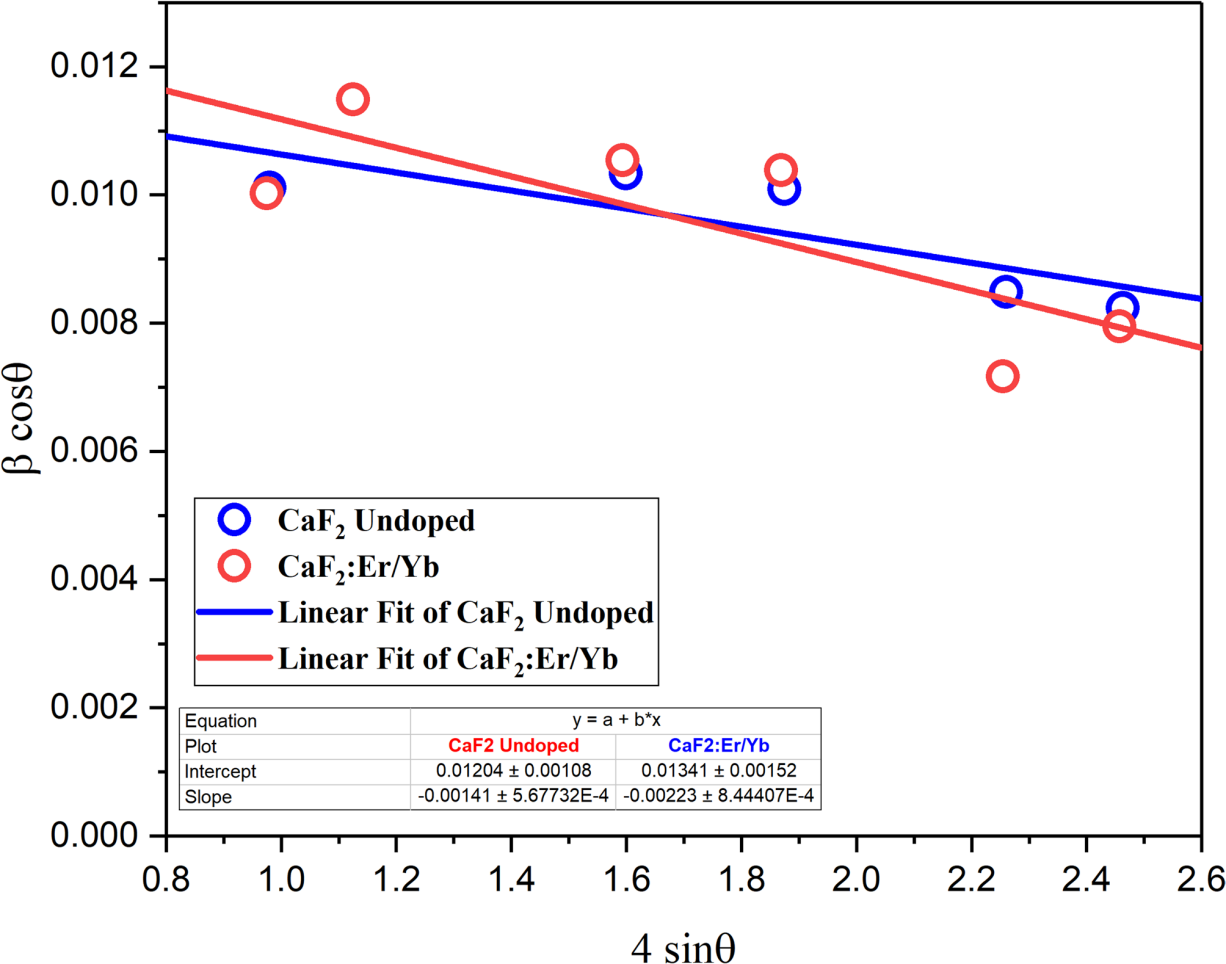
Williamson-Hall plots of undoped CaF_2_ NPs and CaF_2_: Er^3+^/Yb^3+^ UCNPs.

Figure 4 depicts the W-H plot between 4 sin θ vs. β cos θ for undoped CaF_2_ and Er/Yb-doped CaF_2_ with the corresponding linear fit of the data. The slope of the linear fit gives microstrain (ε), and the intercept gives the kλ/D value. The calculated crystallite size of undoped CaF_2_ and Er/Yb-doped CaF_2_ was found to be 12 nm and 10 nm, respectively.Furthermore, thecompressive lattice microstrain of undoped CaF_2_ and Er/Yb-doped CaF_2_ was found to be – 1.41 × 10^−3^and – 2.23 × 10^−3^,respectively. The type of lattice strain may affect the efficiency of the upconversion luminescence^19^. Previously, Ghosh et al.^20^ reported that the upconversion luminescence efficiency was higher in compressive strain than in tensile strain samples.

### Fourier transform infrared (FTIR) spectroscopy

**Fig. 5:**
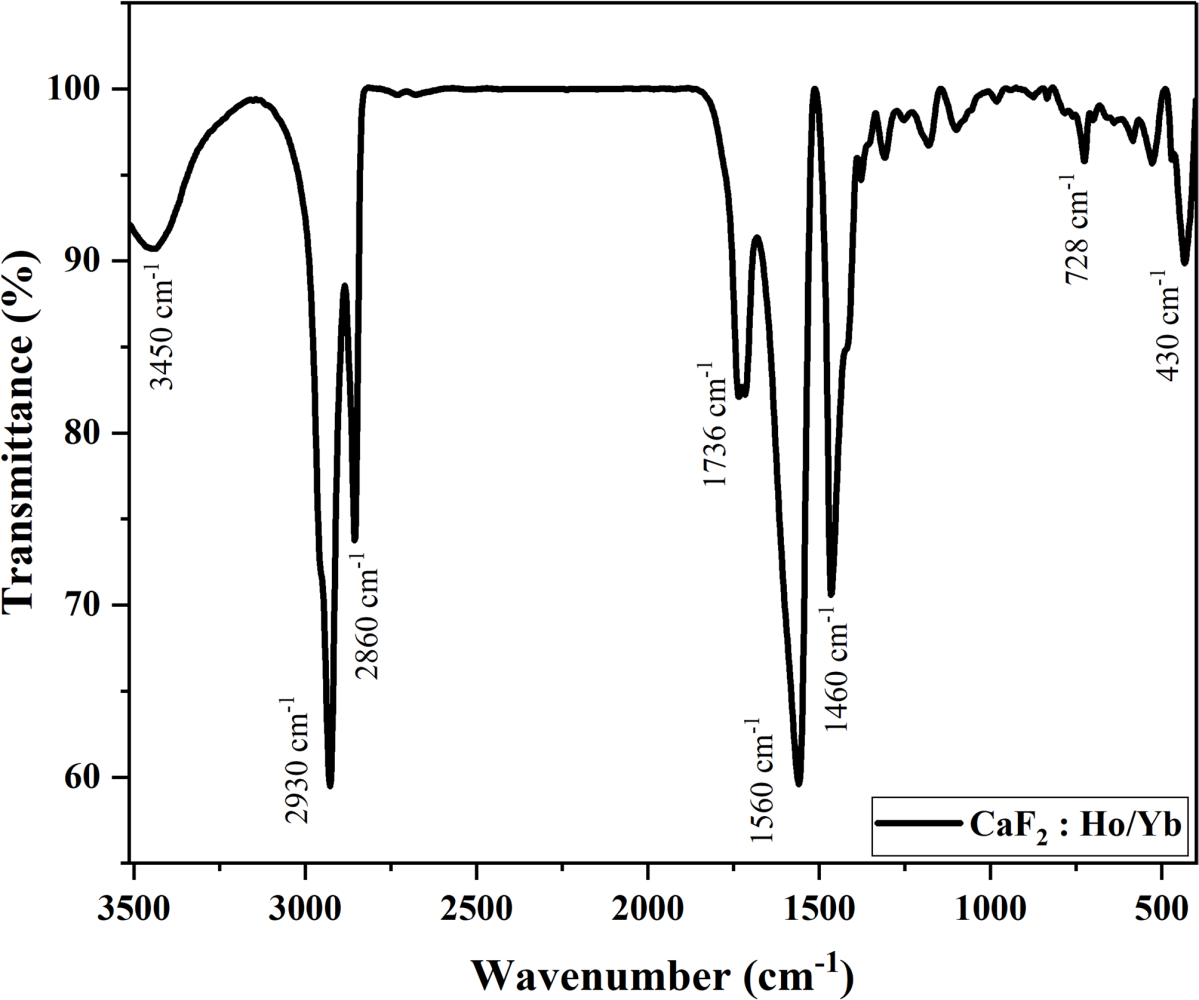
FTIR spectrum of CaF_2_: Ho^3+^/Yb^3+^ UCNPs.

The possible organic surface molecules and chemical bonds present in the sample are studied using FTIR spectroscopy. Figure 5. shows the FTIR spectrum of CaF_2_: Ho^3+^/Yb^3+^UCNPs in the 3510 cm^−1^ to 400 cm^−1^ range. The absorption peak around 3450 cm^−1^ corresponds to the O–H stretching vibration from water. The absorption peaks around 2930 cm^−1^ and 2860 cm^−1^correspond to the anti-symmetric and symmetric stretching of –CH_2_ groups, respectively, from oleic acid. The absorption band around 1736 cm^−1^ arises due to the C = O stretching vibration of the carboxyl group, which is the characteristic peak of free oleic acid. The absorption bands around 1560 cm^−1^ and 1460 cm^−1^ correspond to asymmetric and symmetric stretching vibrations of carboxylate (COO-) groups of oleic acid with UCNPs. The absorption peak around 728 cm^−1^ in the fingerprint region is due to the swing in-planer of the long alkyl chain (CH_2_)_n_ (where *n* > 4). This confirms that the oleic acid is capping the UCNPs^21–24^. The absorption peak at 430 cm^−1^ is associated with the Ca–F stretching vibration of the CaF_2_ host^25^.

### TEManalysis

**Fig. 6:**
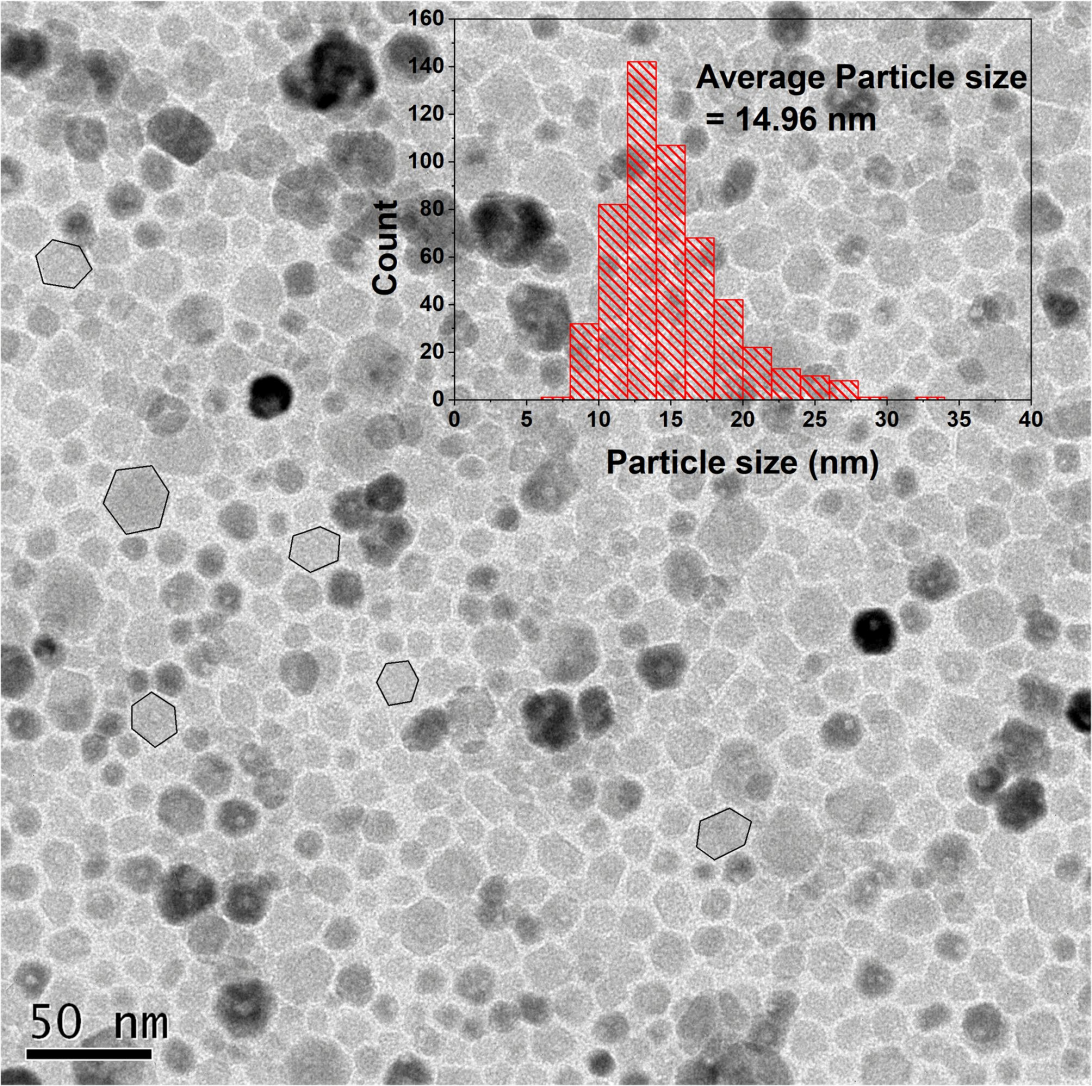
TEM image of CaF_2_ NPs. The inset shows the particle size distribution of NPs.

**Fig. 7:**
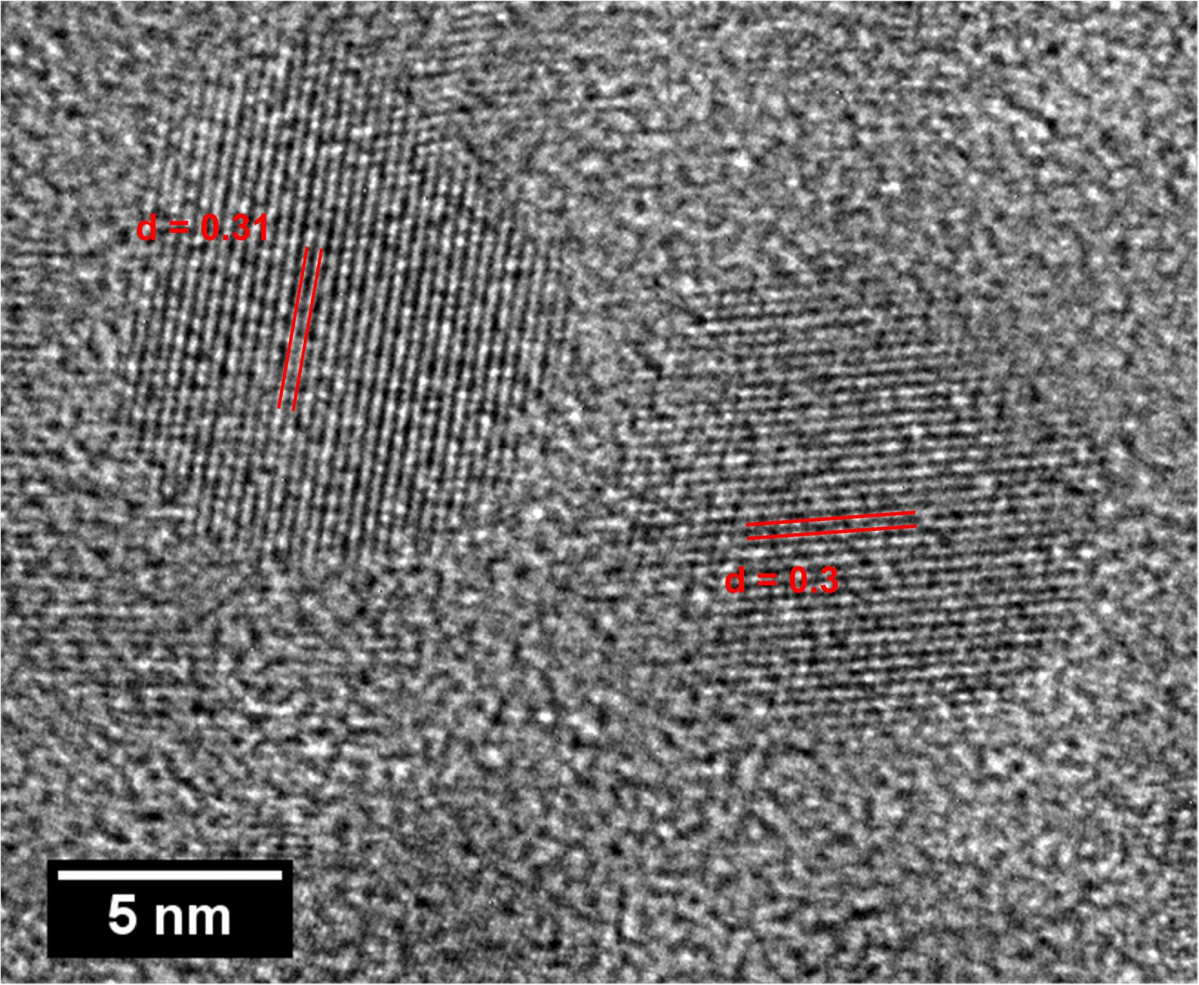
HR-TEM image of CaF_2_ NPs.

**Fig. 8:**
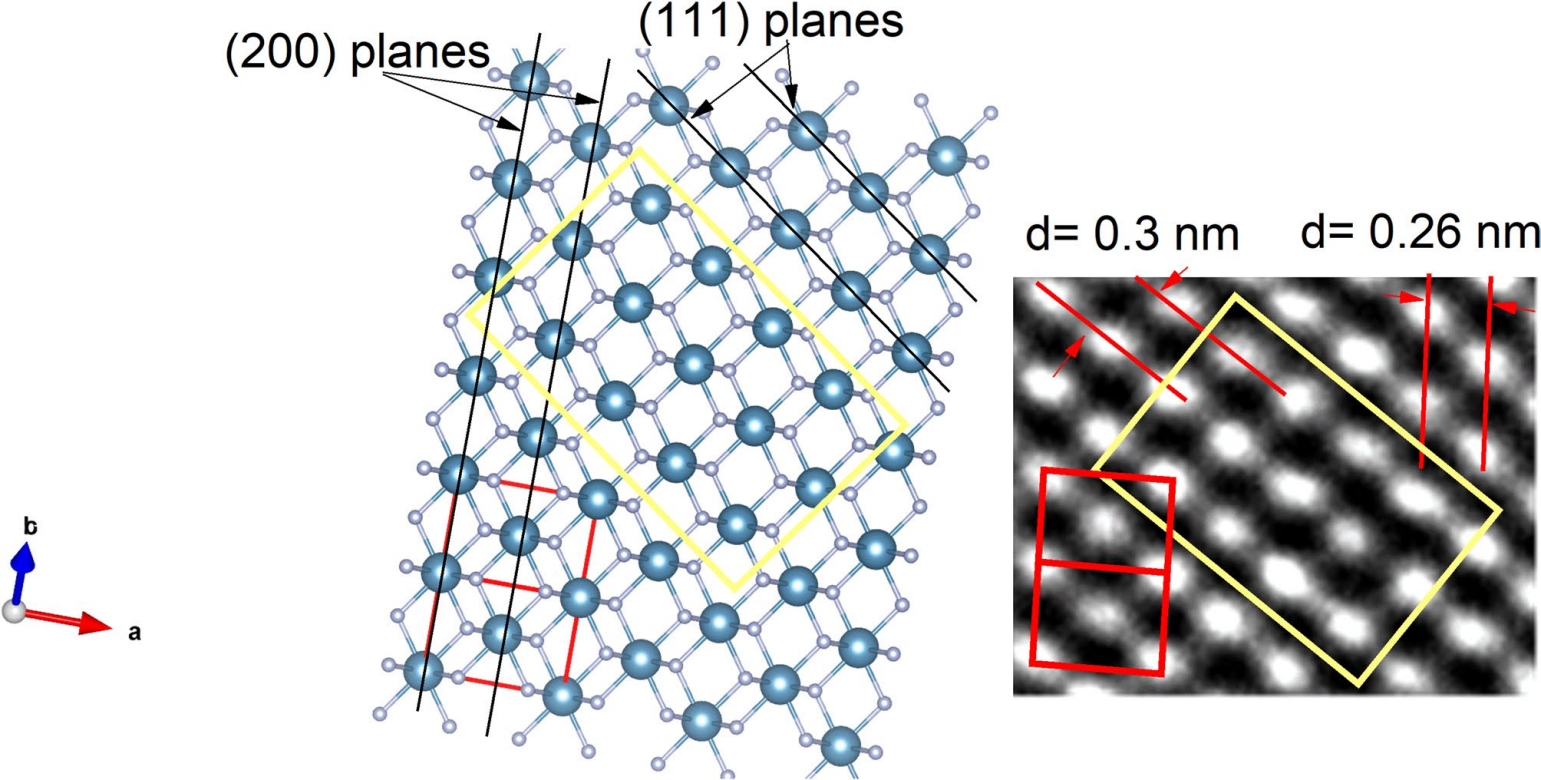
HR-TEM image with corresponding crystal structure of CaF_2_ NPs.

**Fig. 9:**
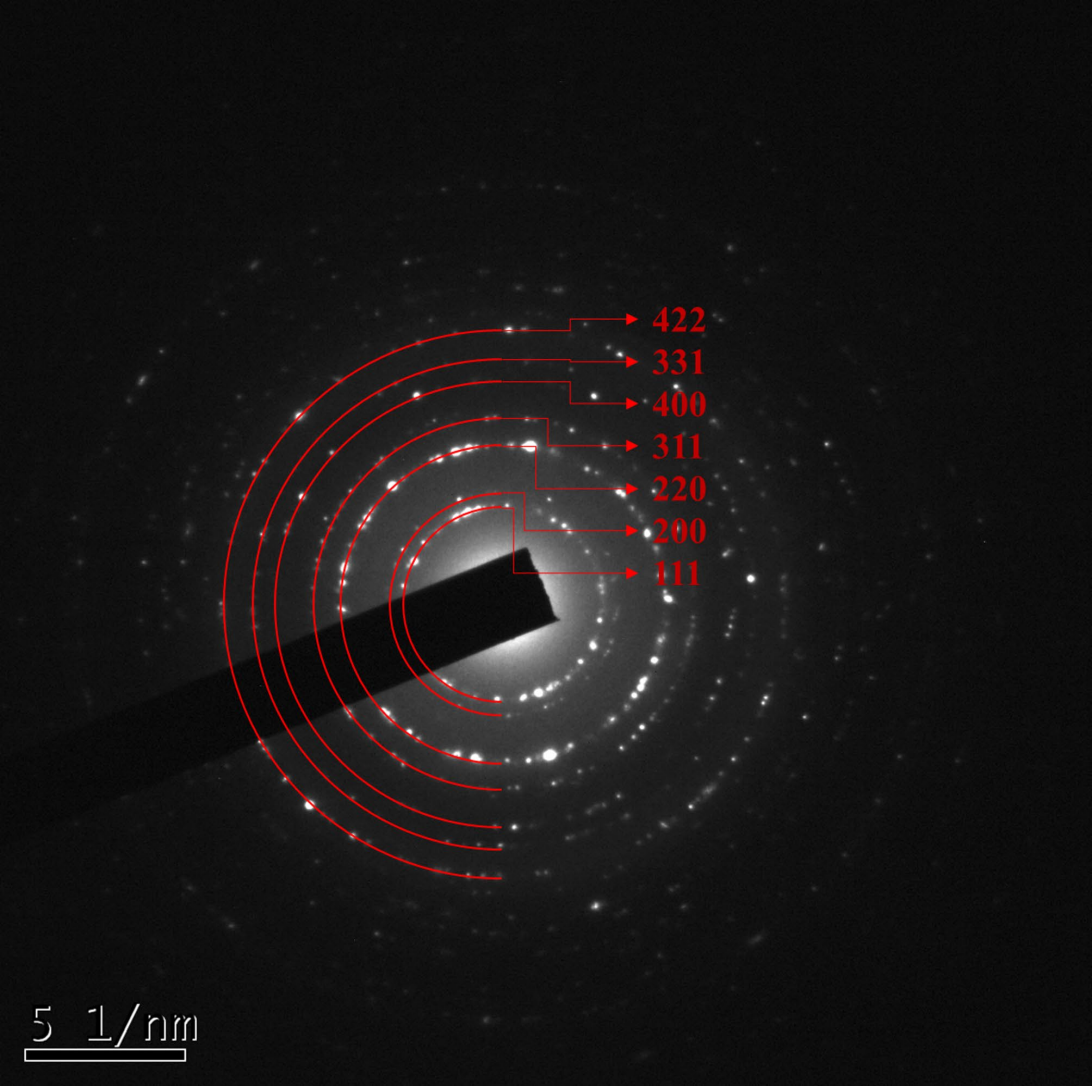
SAED patterns of CaF_2_ NPs.

The transmission electron microscope (TEM) image (Fig. 6.) shows that the sample consists of monodispersed spherical and hexagonal-shaped CaF_2_ Nanoparticles. The inset image shows a particle size distribution plot, which indicates that particle sizes range from 6 nm to 30 nm, with an average particle size of 15 nm. This average particle size is close to the average crystallite size calculated from XRD analysis, which suggests the formation of single-crystal nanoparticles. The HR-TEM image (Fig. 7.) shows the lattice planes (111) corresponding to d = 0.31 nm, indicating the crystalline nature of the nanoparticles. Figure 8. shows the HRTEM image with the corresponding crystal structure orientation. The selected area electron diffraction (SAED) pattern, with the corresponding (hkl) planes indexed as (111), (200), (220), (311), (400), (331), and (422), confirms the standard cubic fluorite CaF_2_ lattice structure, asshown in Fig. 9. The SAEDpattern exhibitsdot-ring features, corresponding to the ensembles of single-crystallinenanoparticles^26^.

### Formation mechanism of nanoparticles by liquid-solid-solution (LSS) approach

**Fig. 10:**
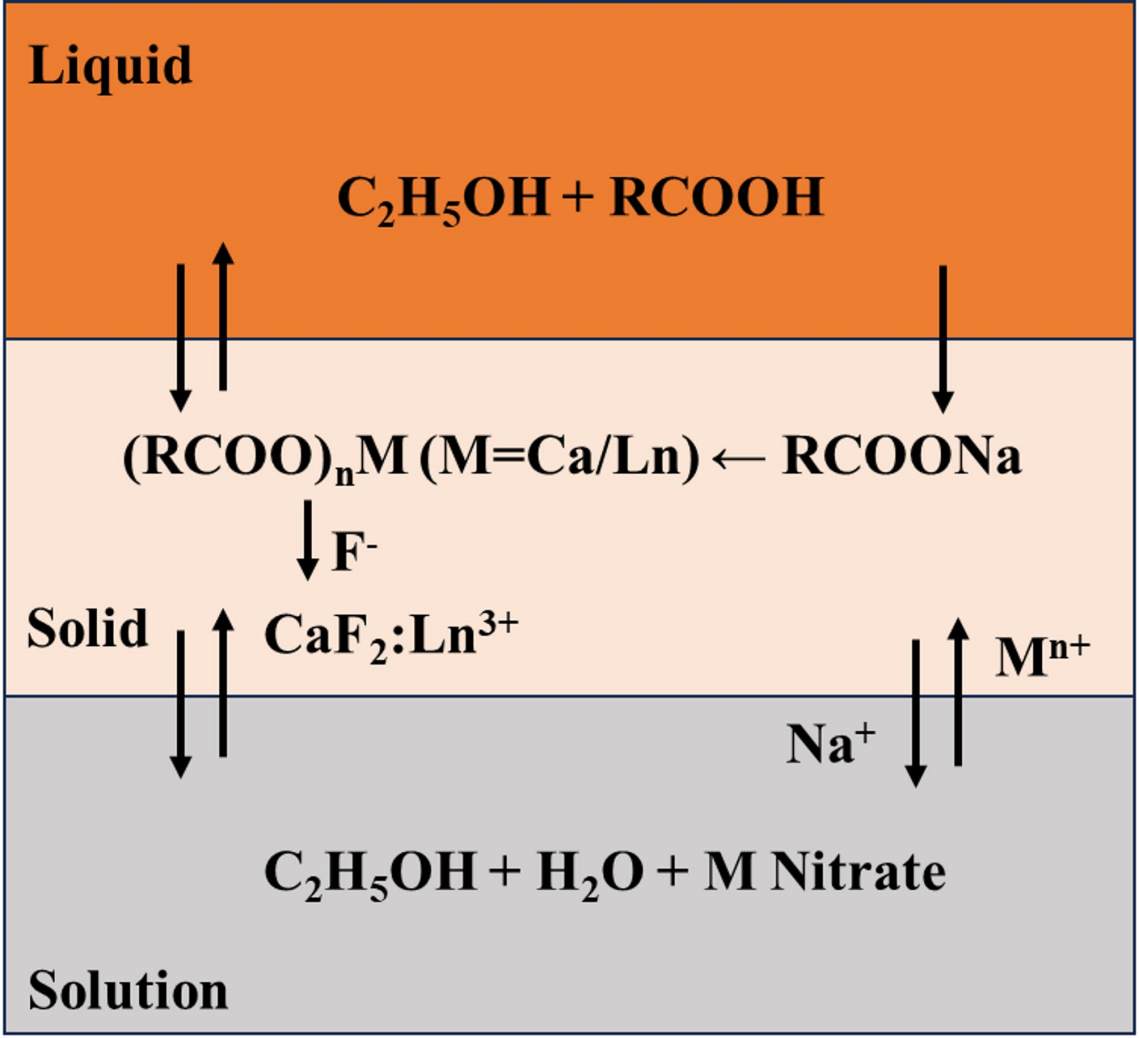
Phase-transfer mechanism in the synthesis of CaF_2_: Ln^3+^ NPs by liquid-solid-solution approach.

The synthesis method of lanthanide-doped CaF_2_ nanoparticles is mentioned in the experimental section in detail. The formation mechanism of MF_2_ (M = Ca, Sr, Ba) nanoparticles by the LSS approach was reported previously^24^. The synthesis involves phase transfer between the liquid phase, the solid phase, and the solution phase in the formation of nanoparticles. Figure 10. shows the LSS phase transfer synthesis mechanism for CaF_2_nanoparticles. First, oleic acid, ethanol, and NaOH mixed form a two-phase mixture; the first is a liquid phase consisting of oleic acid and ethanol, and the second is a solid phase comprisingsodium oleate formed by the reaction of oleic acid and NaOH. After that, the metal nitrates, i.e.,the solutions of calcium nitrate and rare earth nitrate, are added, whichform a third phase, i.e., the solution phase. Ethanol–oleic acid–water forms a reverse micelle–microemulsion system. The ion exchange process occurs between sodium oleate from the solid phase and metal ions from the solution phase, which forms the metal oleate in the solid phase. Further, on the addition of NaF solution, the fluoride ions from the solution phase react with the metal oleate to produce the CaF_2_ nanoparticles. The high-temperature and high-pressure solvothermal treatment in an autoclave helps crystallize UCNPs. The oleic acid controls the growth process by capping onto the surface of nanoparticles and preventing further agglomeration of nanoparticles.

### Raman analysis

**Fig. 11:**
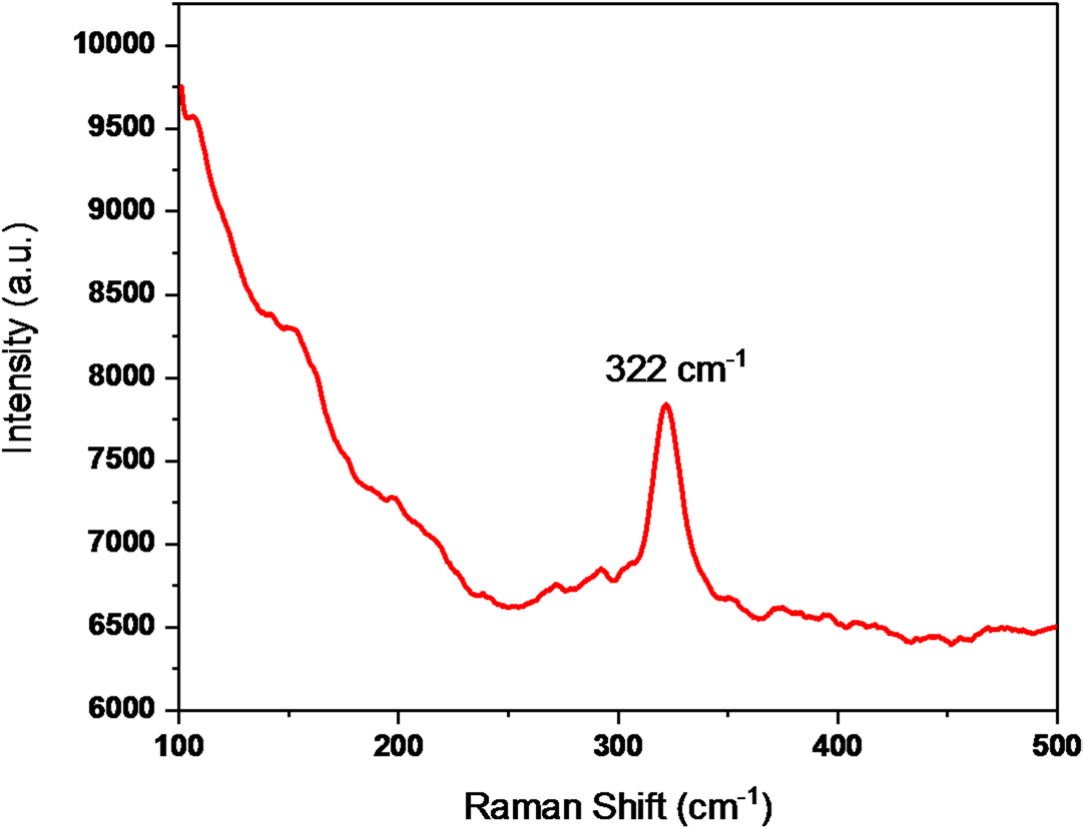
Laser Raman spectrum of undoped CaF_2_ NPs.

Figure 11 shows laser Raman spectra of pure CaF_2_ nanoparticles recorded using a 532 nm laser to determine the phonon energy. The phonon energy plays an important role in upconversion luminescence, as it minimizes the non-radiative losses and improves the quantum efficiency of upconversion. Raman spectra show a peakat 322 cm^−1^,which indicates lower phonon energy than the well-known upconversion host NaYF_4_ (362 cm^−1^)^27^. Thus, CaF_2_ can be considered as a host matrix for upconversion luminescence.

### Photoluminescencespectra

**Fig. 12:**
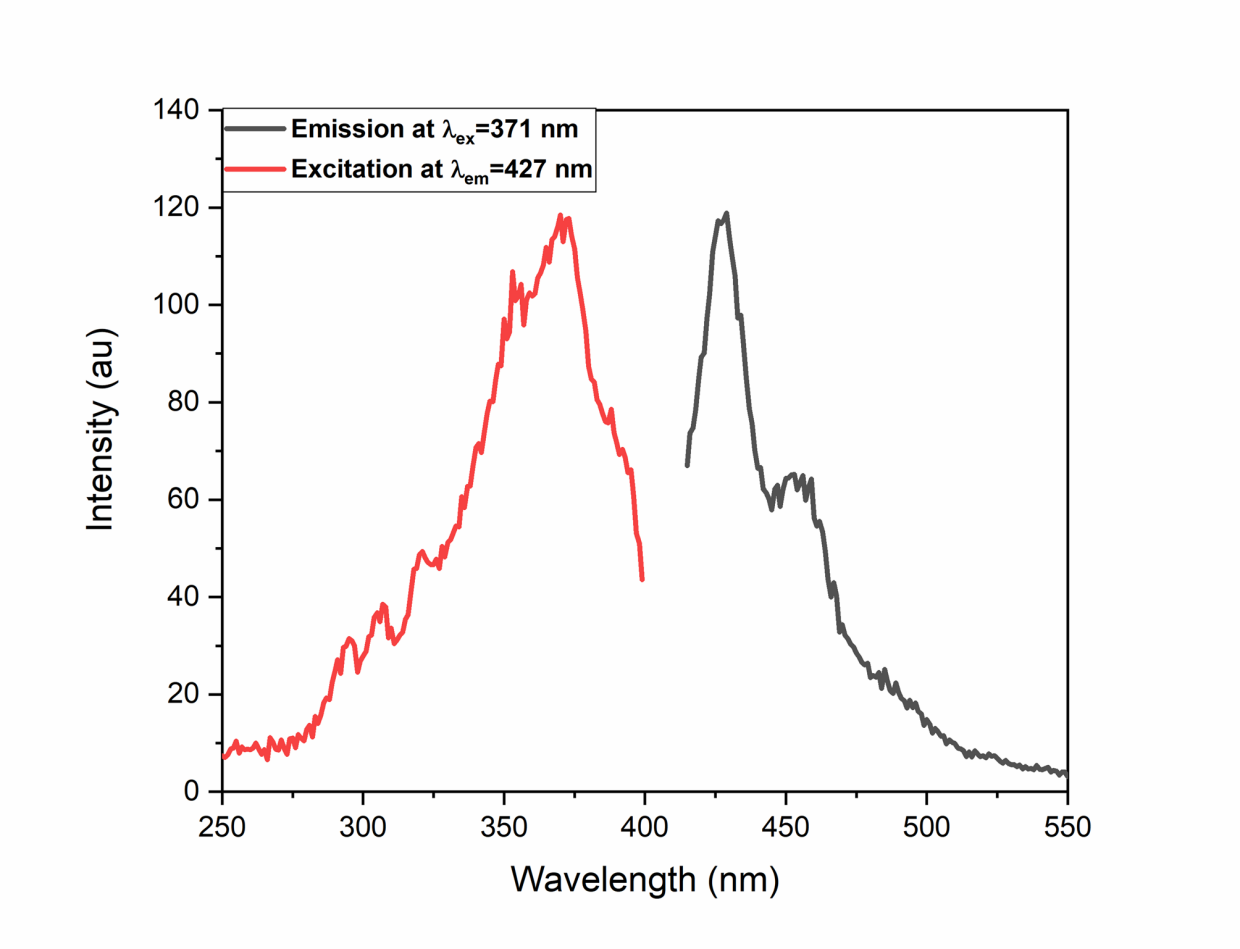
Photoluminescence spectrum of undoped CaF_2_ NPs.

The OA-capped pure CaF_2_ nanoparticles dispersed in cyclohexane were used to study the luminescence properties of the host matrix. Figure 12. shows the photoluminescence properties of undoped CaF_2_ nanoparticles. The excitation spectra show a broad excitation peak maximum around 371 nm when the excitation wavelength is at 427 nm. The emission spectra show an emission peak maximum around 427 nm and a shoulder around 450 nm when excited by 371 nm excitation wavelength. This self-luminescent property may be due to surface defects or electronic centers in the nanoparticles^24^. Hence, the CaF_2_ can be a good host for luminescent and optical materials^28^.

### Upconversion luminescence analysis

**Fig. 13:**
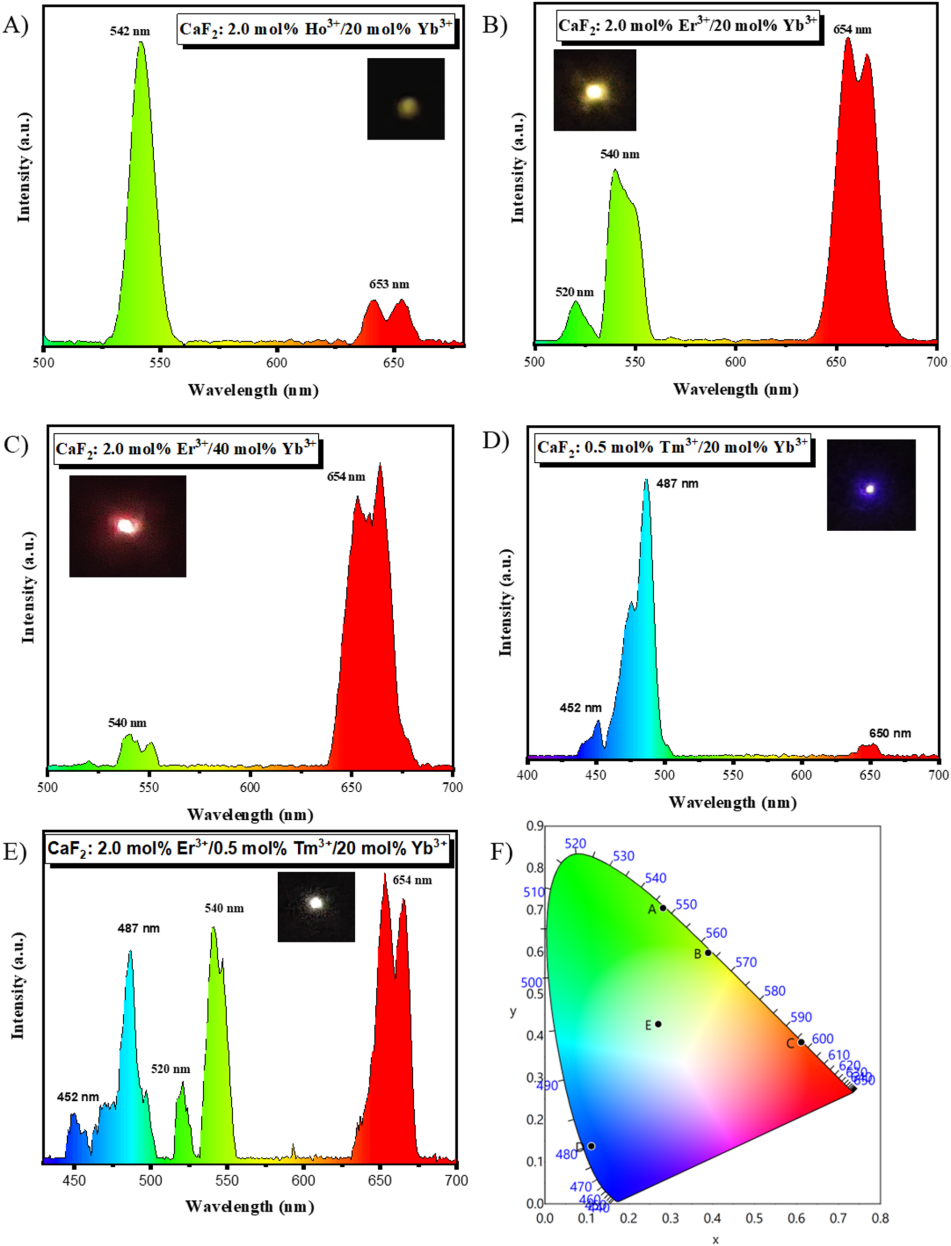
**(A-E)** Upconversion luminescence spectra of **(A)**CaF_2_: 2 mol% Ho^3+^/20 mol% Yb^3+^**(B)** CaF_2_: 2 mol% Er^3+^/20 mol% Yb^3+^**(C)** CaF_2_: 2 mol% Er^3+^/40 mol% Yb^3+^**(D)** CaF_2_: 0.5 mol% Tm^3+^/20 mol% Yb^3+^(**E)** CaF_2_: 2 mol% Er^3+^/0.5 mol% Tm^3+^/20 mol% Yb^3+^ UCNPs recorded under 980 nm, 4 mW/cm^2^ laser excitation. The inset images show the UC glow of corresponding samples under 5 mW laser excitation. (**F)** CIE chromaticity diagram of UCNPs, indicating emission color coordinates of samples **A**-**E**, represented by symbols matchingthose in the corresponding spectrum (**A**-**E**).

**Fig. 14:**
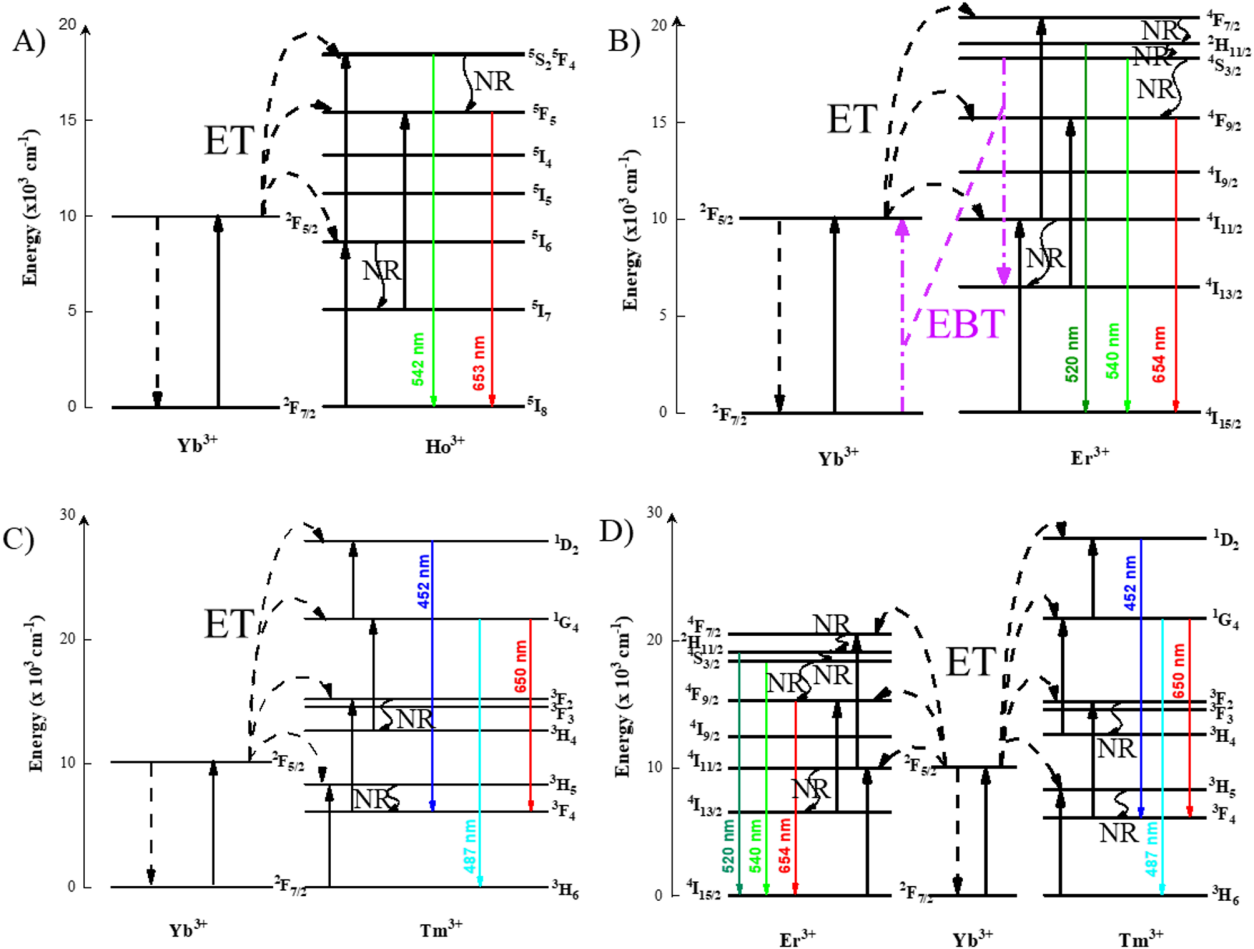
Energy transfer scheme in CaF_2_ UCNPs for **(A)** Ho^3+^/Yb^3+^**(B)** Er^3+^/Yb^3+^**(C)** Tm^3+^/Yb^3+^**(D)** Er^3+^/Ho^3+^/Yb^3+^ co-doped systems. (ET: energy transfer process, NR: Non-radiative relaxation, and EBT: energy back transfer process).

Figure 13. A) shows the upconversion luminescence spectrum of CaF_2_: 2 mol% Ho^3+^/20 mol% Yb^3+^ UCNPs on 980 nm laser excitation. The spectrumshows characteristic emission peaks of Ho^3+^ activator ions. Upconversion spectra showa high-intensity green emission peak at 542 nm and weak red emission peaks inthe 635to 662 nm range of Ho^3+^ ions. This green emission correspondsto the(^5^F_4_/^5^S_2_) →^5^I_8_ transition, and the red emission corresponds to the^5^F_5_ →^5^I_8_transition^29^. This high-frequency visible emission from low-frequency NIR (980 nm) excitation can be explained based on the multi-photon absorption via an energy transfer mechanism, as shown in Fig. 14.A). The Yb^3+^ ions absorb the 980 nm photon to excite from ^2^F_7/2_, ground state to ^2^F_5/2_, excited state via the ground state absorption (GSA) process. This excited Yb^3+^ ion transfers its energy to the Ho^3+^ ions and relaxes to its ground state. First, Yb^3+^transfers its energy to the^5^I_6_ level of Ho^3+^ ions by phonon-assisted non-radiative energy transfer. The energy transfer process can be represented as:^2^F_5/2_ (Yb^3+^) + ^5^I_8_ (Ho^3+^) → ^2^F_7/2_ (Yb^3+^) + ^5^I_6_ (Ho^3+^). The probability of this energy transfer is low since the phonon energy of the host is low; however, once the^5^I_6_ level is populated, further population of higher energy levels is efficient due to the low probability of non-radiative relaxation. Populated^5^I_6_ level can be further excited to the higher ^5^F_4_/^5^S_2_either by directly absorbing a photon from a laser source by the excited state absorption (ESA) process as^5^:I_6_ (Ho^3+^) + photon → ^5^F_4_/^5^S_2_ (Ho^3+^) or by energy transfer from Yb^3+^ as: ^2^F_5/2_ (Yb^3+^) + ^5^I_6_ (Ho^3+^) → ^2^F_7/2_ (Yb^3+^) + ^5^F_4_/^5^S_2_ (Ho^3+^). Thus, the (^5^F_4_/^5^S_2_) →^5^I_8_ transition, which results in 542 nm emission, can be achieved. Furthermore, the^5^F_5_ energy level of Ho^3+^ ions can be populated by non-radiative relaxation (NR) from ^5^F_4_/^5^S_2_. However, there is another pathway to populate the^5^F_5_ energy level, i.e.,the first NR process from the^5^I_6_ to the^5^I_7_energy level, then either by the ESA process as^5^:I_7_ (Ho^3+^) + photon →^5^F_5_ (Ho^3+^) or by energy transfer from excited Yb^3+^ ions as: ^2^F_5/2_ (Yb^3+^) + ^5^I_7_ (Ho^3+^) → ^2^F_7/2_ (Yb^3+^) + ^5^F_5_ (Ho^3+^). Thus, the^5^F_5_ →^5^I_8_transition, which results in 653 nm emission, can be achieved. Note that the 653 nm emission requires the NR process to populate^5^F_5_ in either pathway; therefore, the low emission intensity of the red emission could be affected due to the low probability of NR^30–32^.

Upconversion luminescence spectra of CaF_2_: x Er^3+^/y Yb^3+^ (x = 2 mol% and y = 20 mol%/40 mol%) UCNPs recorded using 980 nm laser excitation. Figure 13. B, C).shows upconversion spectraof CaF_2_: 2 mol% Er^3+^ with varying Yb^3+^ concentrations Fig. 13. B). Yb^3+^ = 20 mol% and Fig. 13. C). Yb^3+^ = 40 mol%. The characteristic peaks of Er^3+^ ions are observed at 520 nm, 540 nm, and 654 nm. The green emission at 520 nm and 540 nm corresponds to the^2^H_11/2_→ ^4^I_15/2_ and^4^S_3/2_ → ^4^I_15/2_transitions, respectively, and the red emission around 654 nm corresponds to the^4^F_9/2_→ ^4^I_15/2_transition^33,34^. The red-to-green emission intensity ratio increased withthe increase inYb^3+^ ion concentration from 20 mol% to 40 mol%. The upconversion mechanism of the Er^3+^/Yb^3+^ co-doped system is studied using the energy transfer diagram as shown in Fig. 14. B). The upconversion process is governed by the ESA process in Er^3+^ ions and energy transfer (ETU) from Yb^3+^to Er^3+^ions. For ESA, the Er^3+^ ions successively absorb sequential photons; first, Er^3+^ populates its ^4^I_11/2_energy level from the ^4^I_15/2_ ground state energy level via the GSA process as:^4^I_15/2_(Er^3+^) + photon → ^4^I_11/2_ (Er^3+^). The ^4^F_7/2_level is populated by further absorption of photons via the ESA process from the^4^I_11/2_ excited state energy level as:^4^I_11/2_(Er^3+^) + photon → ^4^F_7/2_(Er^3+^). The populated ^4^I_11/2_energy level can also undergo the NR process and populate the ^4^I_13/2_ level, which, upon further ESA process, can be populated to the^4^F_9/2_ state energy levelas:^4^I_13/2_(Er^3+^) + photon → ^4^F_9/2_(Er^3+^). For the ETU process, Yb^3+^ ions absorba photon by the GSA process andpopulate the^2^F_5/2_level from the^2^F_7/2_ground state energy level. The Yb^3+^ ions populated at the^2^F_5/2_level transfer their energy to Er^3+^ ions and populate the ^4^I_11/2_energy level from the^4^I_15/2_energy level as:^2^F_5/2_ (Yb^3+^) + ^4^I_15/2_(Er^3+^) → ^2^F_7/2_ (Yb^3+^) + ^4^I_11/2_(Er^3+^), the^4^F_7/2_energy level fromthe^4^I_11/2_energy level as:^2^F_5/2_ (Yb^3+^) + ^4^I_11/2_(Er^3+^) → ^2^F_7/2_ (Yb^3+^) + ^4^F_7/2_(Er^3+^), and the^4^F_9/2_energy level fromthe^4^I_13/2_energy level as:^2^F_5/2_ (Yb^3+^) + ^4^I_13/2_(Er^3+^) → ^2^F_7/2_ (Yb^3+^) + ^4^F_9/2_(Er^3+^) by the ET process. These ESA and ETU processes can work in tandem to populate the Er^3+^ ions via multi-photon absorption processes. The populated ^4^F_7/2_ state can undergo NR processes to occupy ^2^H_11/2_, ^4^S_3/2_, and ^4^F_9/2_ energy levels. These ^2^H_11/2_, ^4^S_3/2_, and ^4^F_9/2_states are the UC emission energy levels, resulting in 520 nm, 540 nm, and 654 nm emissions via radiative transitions, respectively. On increasing the concentration of Yb^3+^ ions from 20 mol% to 40 mol%, the energy back transfer (EBT) process dominates, which means Er^3+^ ions transfer the energy back to the Yb^3+^ ions. In this process, Er^3+^ ions populate the ^4^I_13/2_level from the ^4^S_3/2_energy level by exciting the ground state Yb^3+^ ions to their^2^F_5/2_level. The EBT process can be represented as:^4^S_3/2_(Er^3+^) + ^2^F_7/2_ (Yb^3+^) → ^4^I_13/2_ (Er^3+^) + ^2^F_5/2_ (Yb^3+^). ^4^I_13/2_ level on further ESA, populate the ^4^F_9/2_energy level, which is responsible for red emission. Therefore, increasing the Yb^3+^ concentration enhances the population ofthe^4^F_9/2_energy level (starting from the populated ^4^S_3/2_ state) via the EBT pathway, ultimately boosting the red UC emission intensity of the UCNPs^35–40^.

Figure 13. D) depicts the upconversion luminescence spectrum of CaF_2_: 0.5 mol% Tm^3+^/20 mol% Yb^3+^ UCNPs upon 980 nm laser excitation. The spectrum shows a strong emission band around 487 nm and two weak emission bands around 452 nm and 650 nm. The blue emission band around 487 nm corresponds to the^1^G_4_→^3^H_6_ transition, another blue emission band around 452 nm corresponds to the^1^D_2_→^3^F_4_ transition, and a red emission band around 650 nm corresponds to the^1^G_4_→^3^F_4_ transition of Tm^3+^ ions^41,42^. These characteristic emissions of Tm^3+^ ions are observed due to the ETU process from sensitizer Yb^3+^ ions. Figure 14. C).shows the energy transfer scheme for energy transfer from Yb^3+^ to Tm^3+^ ions. The Yb^3+^ ions in their ground state absorb a photon and excite to the ^2^F_5/2_ level via the GSA process as discussed in previous systems, and transfer their energy to Tm^3+^ ions to populate various energy levels. Tm^3+^ ions populate to the^3^H_5_ energy level as: ^2^F_5/2_ (Yb^3+^) + ^3^H_6_ (Tm^3+^) → ^2^F_7/2_ (Yb^3+^) + ^3^H_5_ (Tm^3+^) from the^3^H_6_ energy level, the^3^F_2_ energy level as:^2^F_5/2_ (Yb^3+^) + ^3^F_4_ (Tm^3+^) → ^2^F_7/2_ (Yb^3+^) + ^3^F_2_ (Tm^3+^) from the^3^F_4_ energy level, the^1^G_4_ energy level as:^2^F_5/2_ (Yb^3+^) + ^3^H_4_ (Tm^3+^) → ^2^F_7/2_ (Yb^3+^) + ^1^G_4_ (Tm^3+^) from the^3^H_4_ energy level, and the^1^D_2_ energy level as:^2^F_5/2_ (Yb^3+^) + ^1^G_4_ (Tm^3+^) → ^2^F_7/2_ (Yb^3+^) + ^1^D_2_ (Tm^3+^) from the^1^G_4_ energy levelvia energy transfer from Yb^3+^ ions. The^3^F_4_ and^3^H_4_ energy levels are populated by the NR process from the^3^H_5_ and^3^F_2_ energy levels, respectively. The populated^1^D_2_ energy level can lead to emission of 452 nm photons, whereas the^1^G_4_ level can lead to emission of 487 nm and 650 nm photons^43,44^.

The luminescence spectra of CaF_2_: 2 mol% Er^3+^/20 mol% Yb^3+^ and CaF_2_: 0.5 mol% Tm^3+^/20 mol% Yb^3+^ UCNPs indicatepromising potentialfor white light generation. This motivated the synthesis of CaF_2_: 2 mol% Er^3+^/0.5 mol% Tm^3+^/20 mol% Yb^3+^ UCNPs. The CaF_2_: 2 mol% Er^3+^/0.5 mol% Tm^3+^/20 mol% Yb^3+^UCNPs were evaluated for white light generation capabilities upon NIR laser excitation. Figure 13. E). shows the upconversion luminescence spectrum of CaF_2_: Er^3+^/Tm^3+^/Yb^3+^ UCNPs. The upconversion emission spectrum shows distinct bands at 452 nm, 487 nm, 520 nm, 540 nm, and 654 nm, corresponding to the^1^D_2_→^3^F_4_ and^1^G_4_→^3^H_6_transitions of Tm^3+^ions, as well as the ^2^H_11/2_→ ^4^I_15/2_, ^4^S_3/2_ → ^4^I_15/2_, and ^4^F_9/2_→ ^4^I_15/2_transitions of Er^3+^ ions, respectively. These emissions confirm the simultaneous activation of Er^3+^ and Tm^3+^ activator ions in the presence of Yb^3+^ sensitizer ions^45^. The schematic energy transfer diagram of the Er^3+^/Tm^3+^/Yb^3+^ triply doped system is shown in Fig. 14. D). The mechanism of energy transfer from Yb^3+^ to Er^3+^ and Yb^3+^ to Tm^3+^, leading to the population of respective excited energy states, has been discussed earlier. The observed emission bands validate the successful co-doping of Er^3+^ and Tm^3+^activator ions along with the Yb^3+^ sensitizer ions for the spectral output in white light generation.

Figure 13. F). shows the Commission Internationale de l’Éclairage(CIE-1931) chromaticity coordinates of the CaF_2_ UCNPs withvarious dopant systems, calculated using the corresponding upconversion emission spectra.UCNPs, their chromaticity coordinates, and color purity are summarized in Table 1. CaF_2_: 2 mol% Ho^3+^/20 mol% Yb^3+^ UCNPs show highly pure green emission. CaF_2_: 2 mol% Er^3+^/20 mol% Yb^3+^ UCNPs show yellow-greenish emission, whichupon increasing Yb^3+^ concentration, i.e., CaF_2_: 2 mol% Er^3+^/40 mol% Yb^3+^ UCNPs show red emission. CaF_2_: 0.5 mol% Tm^3+^/20 mol% Yb^3+^ UCNPs show blue emission. CaF_2_: 2 mol% Er^3+^/0.5 mol% Tm^3+^/20 mol% Yb^3+^ UCNPs fall within the white-light region in the CIE plot.

**Table 1:** CIE-1931 chromaticity coordinates and color purity of CaF_2_ ucnps.

Symbol	Sample composition	CIE Color coordinates	Color purity
A	CaF_2_: 2 mol% Ho^3+^/20 mol% Yb^3+^	(0.282, 0.705)	98.3%
B	CaF_2_: 2 mol% Er^3+^/20 mol% Yb^3+^	(0.389, 0.598)	96.8%
C	CaF_2_: 2 mol% Er^3+^/40 mol% Yb^3+^	(0.611, 0.385)	99.0%
D	CaF_2_: 0.5 mol% Tm^3+^/20 Yb^3+^	(0.111, 0.137)	93.4%
E	CaF_2_: 2 mol% Er^3+^/0.5 mol% Tm^3+^/20 mol% Yb^3+^	(0.271, 0.428)	-

### Latent fingerprint detection

**Fig. 15:**
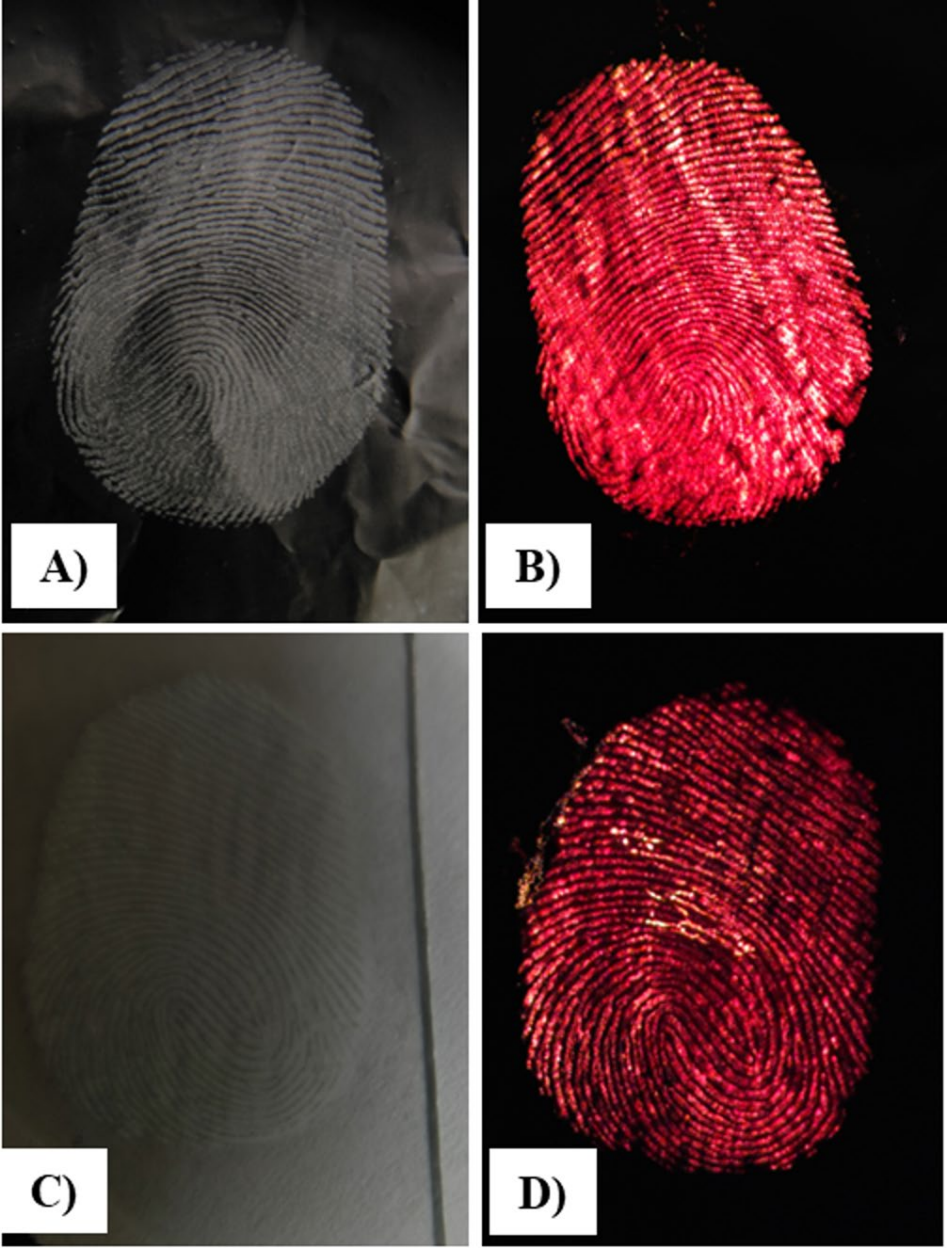
Digital photographs of developed latent fingerprints in **(A**,** C)** daylight and **(B**,** D)** 980 nm laser on two different substrates **(A**,** B)** Aluminium foil and **(C**,** D)** Glass plate.

To determine the scope of the prepared nanoparticles in latent fingerprint detection, the CaF_2_: 2 mol% Er^3+^/20 mol% Yb^3+^ UCNPs were selected, and the UC efficiency was enhanced by an annealing process at 600 °C. After annealing, the upconversion emission intensity increased, with a noticeable enhancement in the overall red emission.For the development of latent fingerprints, two different substrates, namely a glass plate and aluminium foil, were selected and treated as described in our previous work^46^for capturing the developed fingerprints. The fingers were first washed thoroughly using soap and water. Then the finger is gentlywiped on the forehead and imprinted by pressing it on the surfaces of the selected substrate. Phosphor powder was then carefully applied to the finger marks, and excess powder was removed by lightly dusting it using a soft brush.The latent fingerprints were visualized by scanning the surface with a 980 nm, 5 mW excitation laser.The images of red UC emission from the fingerprints were captured using a mobile phone camera equipped with an NIR cut filter. Figure 15. shows photographs of fingerprints developed on aluminium foil under daylight (Fig. 15.(A).), aluminium foil under 980 nm excitation (Fig. 15.(B).), a glass plate under daylight (Fig. 15.(C).), and a glass plate under 980 nm excitation (Fig. 15.(D).). The images show strong red emissions from the samples, demonstrating the potential of the UCNPs for latent fingerprint detection.

### Anti-counterfeiting applications

**Fig. 16:**
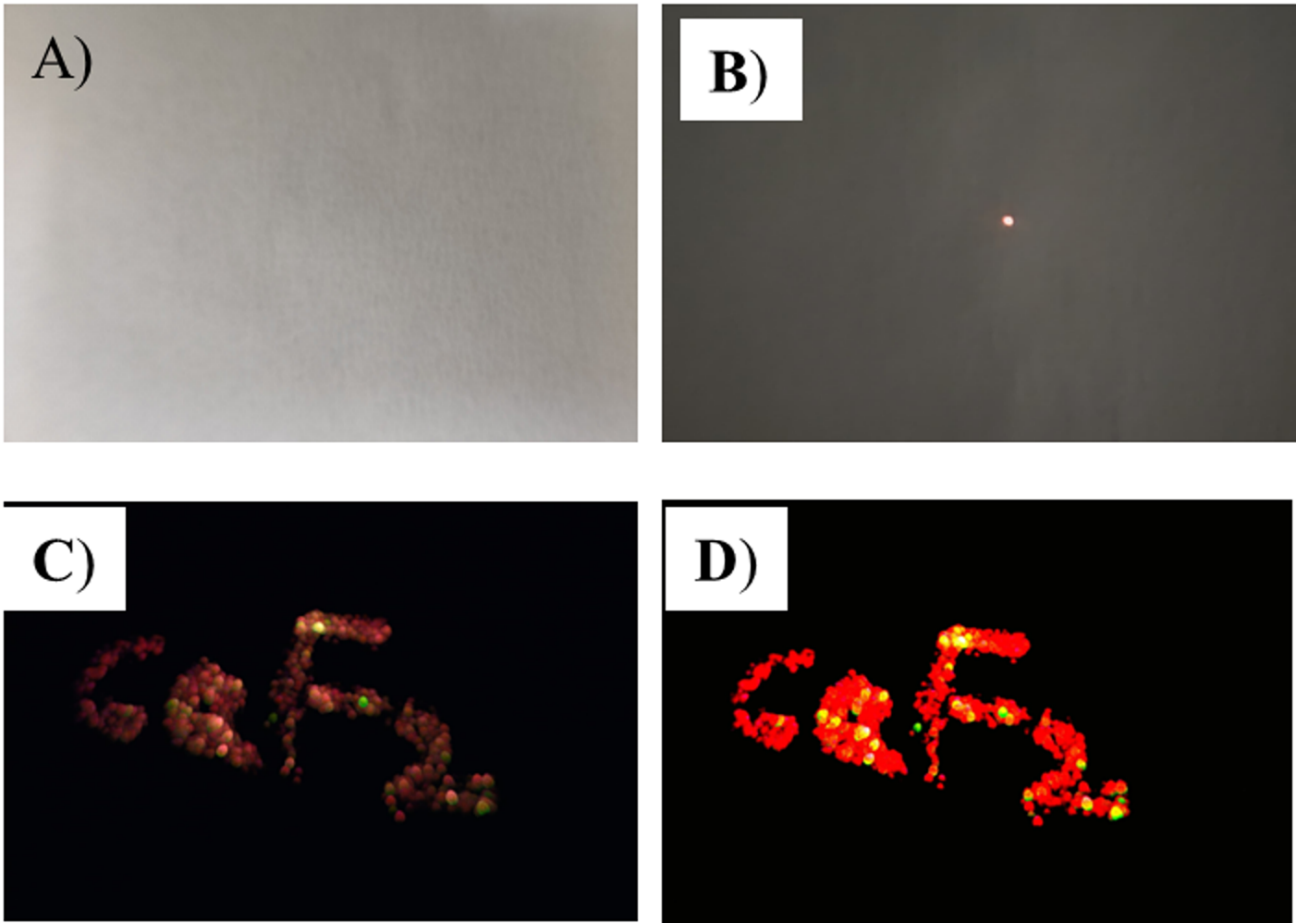
Digital photographs of Anti-counterfeiting letters in **(A)** daylight, **(B)** under 980 nm Laser, **(C)** scanned using 980 nm laser, and **(D)** digitally enhanced image.

For the security writing application, CaF_2_: 2 mol% Er^3+^/40 mol% Yb^3+^OA-capped UCNPs were dispersed in chloroform using ultrasonication in a sonication bath for 15 min. The dispersed UCNPs were used to write the letters “CaF_2_”on 75 GSM A4 paper (JK Copier) manually using a cotton swab, demonstrating a straightforward approach for security applications. Figure 16. shows photographs of hidden letters in daylight and under 980 nm excitation. The letters are invisible in the daylight conditions (Fig. 16. (A).); however, on 980 nm laser excitation, the emission becomes visible (Fig. 16.(B).). The developed letters were visualized by scanning the paper using a 980 nm laser (similar to fingerprint generation) and captured by using a mobile camera equipped withan NIR filter(Fig. 16. (C).), For the enhanced visualization (since a low-power laser was used), the image was further processed digitally by adjusting brightness and contrast to highlight the emission of the patterns(Fig. 16. (D).).

## Conclusion

CaF_2_ UCNPs were successfully synthesized using the solvothermal method by the liquid-solid-solution approach. The XRD patterns are well-matched with standard patterns (ICDD file no. 01-077-2093), which indicate the formation of a cubic fluorite structure. The Rietveld refinement analysis shows an increase in lattice parameters with the doping of lanthanide ions due to the interstitial charge-compensating fluorine ions. Functional groups present in the sample were identified using the FTIR spectrum, which shows the successful capping of oleic acid on the nanoparticles. HR-TEM analysis shows the formation of near-monodispersed spherical and hexagonal-shaped single-crystal nanoparticles with an average particle size of 15 nm. The phonon energy of the host was determined by using laser Raman spectroscopy. The upconversion properties of CaF_2_nanoparticles with double-doping of 2 mol% Ho^3+^/20 mol% Yb^3+^, 2 mol% Er^3+^/20 mol% Yb^3+^, 2 mol% Er^3+^/40 mol% Yb^3+^, 0.5 mol% Tm^3+^/20 mol% Yb^3+^, and triple-doping of 2 mol% Er^3+^/0.5 mol% Tm^3+^/20 mol% Yb^3+^ were investigated. Appropriate doping of lanthanide ions shows green, yellow, red, blue, and white emission under 4 mW/cm^2^ 980 nm laser excitation. The UCNPs with multi-color emissions have potential scope in bioimaging, phototherapy, fluorescent labels, lighting devices, displays, forensic science, and detection applications. The scope of UCNPs was successfully examined for latent fingerprint detection and anticounterfeit applications.

## Data Availability

The datasets used and/or analysed during the current study are available from the corresponding author on reasonable request.
